# Draft Genome Sequence of *Burkholderia* sp. Strain MP-1, a Methyl Parathion (MP)-Degrading Bacterium from MP-Contaminated Soil

**DOI:** 10.1128/genomeA.00344-14

**Published:** 2014-05-22

**Authors:** Xu-Yun Liu, Xiao-Jing Luo, Chun-Xiu Li, Qi-Liang Lai, Jian-He Xu

**Affiliations:** a Laboratory of Biocatalysis and Synthetic Biotechnology, State Key Laboratory of Bioreactor Engineering, East China University of Science and Technology, Shanghai, China; b State Key Laboratory Breeding Base of Marine Genetic Resources, Key Laboratory of Marine Genetic Resources, Third Institute of Oceanography, SOA, Key Laboratory of Marine Genetic Resources of Fujian Province, Xiamen, China

## Abstract

*Burkholderia* sp. strain MP-1 was isolated from pesticide-contaminated soil. Herein, we report the draft genome sequence of strain MP-1, which contains 168 contigs of 8,611,053 bp, with a G+C content of 62.55% and 7,631 protein-coding genes.

## GENOME ANNOUNCEMENT

*Burkholderia* spp. are widely distributed in nature and include a large variety of related environmental, clinical, and agribiotechnological species (1). The genus *Burkholderia* now comprises 78 species (http://www.bacterio.net/burkholderia.html). A methyl parathion (MP)-degrading bacterial strain, designated as *Burkholderia* sp. strain MP-1 (LMG 27927 = MCCC 1K00250), was isolated from the contaminated soil of a former pesticide-manufacturing company in Jiangsu province of China. The 16S rRNA sequence of the newly isolated strain showed the highest similarity to that of *Burkholderia grimmiae* DSM 25160^T^ (98.45%) (2).

The genome sequence of strain MP-1 was determined by Shanghai Majorbio Bio-pharm Technology Co., Ltd. (Shanghai, China), with a MiSeq system using paired-end sequencing technology. A total of 3,758,646 pair reads and 9,573 clean single reads (500-bp library; read length, 300 bp × 2) were assembled using Newbler 2.6. The genome of *Burkholderia* sp. MP-1 contains 168 contigs (>500 bp; *N*_90_, 69) of 8,611,053 bp (131-fold coverage), with an average G+C content of 62.55%. Automatic gene annotation was performed by the NCBI Prokaryotic Genomes Automatic Annotation Pipeline (PGAAP) (http://www.ncbi.nlm.nih.gov/genomes/static/Pipeline.html). The genome comprises 7,791 genes, including 55 tRNA genes for all 20 amino acids, 3 rRNA genes, 1 small noncoding RNA gene, 101 pseudogenes, and 7,631 protein-coding genes (CDS) (with an average length of 950 bp), which give a coding intensity of 84.2%. In all, 4,853 proteins were assigned to Clusters of Orthologous Groups (COG) families (3). Among these proteins, the ones associated with general function prediction (R; 860 open reading frames [ORFs], 17.7%) are the most abundant content of COG, followed by the proteins related to amino acid transport and metabolism (E; 744 ORFs, 15.3%) and transcription (K; 534 ORFs, 11.0%).

Strain MP-1 was able to metabolize MP, a common organophosphorus pesticide employed as the sole carbon source for growth. According to the annotation results, the gene encoding phosphotriesterase exists in the genome. Several draft genomes of *Burkholderia* strains were investigated, such as that of *Burkholderia pyrrocinia* CH-67, whose genome contains genes encoding enzymes for aromatic compound degradation (4). The genome sequence of strain MP-1 and its curated annotation were analyzed to provide abundant genetic information and to explore useful functions.

### Nucleotide sequence accession number.

The draft genome sequence of strain MP-1 has been deposited at GenBank under the accession no. JFHF00000000 (chromosome).
